# Prophylaxis of Venereal Diseases

**Published:** 1882-04

**Authors:** 


### PROPHYLAXIS OF VENEREAL DISEASES.

The session of *Congress of Hygienists*, at Milan, September 3, 1881, discussed the question of the prophylaxis of venereal diseases. The following were the conclusions reached :

1st. Public prophylaxis against the diffusion of syphilis is of such social importance that the obligation of instituting and maintaining it constitutes a duty imperative upon the government itself.

2d. Prostitution being the chief means of diffusion of the syphilitic affections, the government has the right and the duty of keeping watch over the women who devote themselves to this vice, and to submit them to a medical inspection periodically, and at short intervals.

3d. The weekly medical inspection of the soldiers and marines should be scrupulously carried out.

4th. A sanitary inspection should be established for the merchant marine service, for the workmen at ports and arsenals, for the great state manufactories or public departments, for workmen in glass factories, for prisoners of both sexes detained under the imputation of crimes or misdemeanors.

5th. While recommending the erection of special syphilitic hospitals in populous commercial cities, the admission of syphilitics into all the ordinary hospitals should be facilitated.

6th. It was recommended to establish free dispensaries for the treatment of venereal diseases.

7th. In demanding the adoption of a law for the protection of infants, it would be indispensable to establish municipal commissions, which should have oversight of nurses and nurslings, for the purpose of arresting at once congenital syphilis and syphilis by nursing.

8th. To create refuges or asylums for depraved minor women and for repentant prostitutes.

The Congress is of the opinion that we should favor social, economic and educational measures which should deter women from prostitution ; that it is necessary to treat the social wound of prostitution in its causes and in its origin ; that when prostitution shall be made to cease, and especially uncontrolled prostitution, the problem of the extinction of syphilis will become of easy solution.—*La Presse Méd. Belge—St. Louis Courier of Medicine.*

